# Design Considerations for Smoking Cessation Apps: Feedback From Nicotine Dependence Treatment Providers and Smokers

**DOI:** 10.2196/mhealth.5181

**Published:** 2016-02-12

**Authors:** Jennifer B McClure, Andrea L Hartzler, Sheryl L Catz

**Affiliations:** ^1^ Group Health Research Institute Seattle, WA United States; ^2^ Betty Irene Moore School of Nursing University of California, Davis Sacramento, CA United States

**Keywords:** tobacco use cessation, smoking, mobile health, smartphone

## Abstract

**Background:**

Hundreds of smoking cessation apps are commercially available, but most are not theory-based or designed to take advantage of mobile technology in ways that could make them more engaging and possibly more effective. Considering input from both clinical experts (who understand best practice nicotine dependence treatment requirements) to inform appropriate content and from smokers (the end users) to express their preferences is important in designing these programs in the future.

**Objective:**

To assess and compare the opinions of nicotine dependence treatment providers and smokers regarding the design of future smoking cessation apps.

**Methods:**

We surveyed providers (n=264) and smokers who own smartphones (n=40) to assess their opinions on the importance of 21 app design features. Features represented 5 domains: cost, reputation, privacy and security, content and user experience, and communication. Domains were chosen to reflect best practice treatment, leverage mobile technology to support smoking cessation, and elicit important user preferences. Data were collected between June and July 2015.

**Results:**

Most providers agreed that mHealth apps hold promise for helping people quit smoking (203/264, 76.9%) and would recommend them to their clients/patients (201/264, 76.1%), especially if the app were empirically validated (236/264, 89.4%). Few providers believe effective cessation apps currently exist (112/264, 42.4%). Few smokers (5/40, 13%) had ever downloaded a smoking cessation app; of the ones who had not, most said they would consider doing so (29/35, 83%). Both respondent groups indicated the following features were very to extremely important to include in cessation apps: free or low cost, keeps information private, matches individual needs and interests, adapts as one’s needs and interests change, helps to manage nicotine withdrawal symptoms and medication side effects, and allows users to track their progress. Providers and smokers also indicated gaming and social media connectivity were less important than other features. Despite these similarities, the groups had significantly different opinions about the relative importance of various features. In particular, providers rated privacy as the most important feature, whereas smokers rated low cost and the ability to adaptively tailor content as the most important features.

**Conclusions:**

Smoking cessation apps hold great promise as intervention tools but only if they engage users and appropriately treat nicotine dependence. Intervention development should take into consideration the perspectives of both treatment experts and smokers. This paper highlights important perspectives from each of these groups to be considered when designing future app-based smoking cessation programs.

## Introduction

According to the World Health Organization, mobile Health (mHealth) technologies have the potential to transform the face of health service delivery [1]. mHealth interventions, particularly smartphone apps, offer many treatment benefits such the relatively low cost of intervention and wide potential reach. Smartphones are increasingly the device with which lower income and minority populations access the Internet [2], making smartphone apps an important public health tool and an obvious modality for delivering population-based smoking cessation interventions. In the United States alone, 64% of adults own a smartphone [3], and 17% are current smokers [4]. Thus, there is great opportunity to create mHealth tobacco cessation programs.

Other benefits of mHealth smoking cessation programs include accessibility, personalization, and convenience—content can be viewed on-demand 24/7. Content delivered through an app or mobile-enabled website can be dynamically updated in response to user-provided content on changing needs, interests, or situations (eg, nicotine withdrawal symptoms, cravings to smoke, or medication side effects). In addition, mobile apps can capitalize on the strengths of social media to allow interaction with other smokers trying to quit, share knowledge and experiences, and create a sense of community. As a result, cessation apps may be more acceptable and engaging than other population-level interventions such as written materials or tobacco quitlines, resulting in a higher therapeutic exposure and greater impact on treatment outcomes.

To date these benefits are largely hypothetical and untested*.* A recent review of commercially available products concluded that most smoking cessation apps are simplistic and not particularly “smart” [5] in that their designs do not take advantage of the technological capacities of smartphones to do things such as adaptively tailor content or allow 2-way interactions between users or users and clinicians. In fact, most existing commercial cessation apps do not even include best practice treatment. For example, most address only 2 of the 5 A’s (ask, advise, assess, assist, and arrange follow-up) considered to be core aspects of appropriate nicotine dependence intervention [6,7]. Other studies have found that only a handful of publicly available cessation apps recommend the use of approved stop-smoking medications, and none recommend users seek additional counseling from a tobacco quitline [8,9], an evidence-based, free treatment resource available to smokers in almost all US states. In other words, most existing cessation apps seem to have been developed without an understanding of the complexities of nicotine dependence treatment.

Moreover, most apps appear to have been developed without taking into consideration user needs. In fact, few user-centered studies have focused specifically on cessation app design [10,11] as opposed to the design of other mobile-delivered cessation interventions via short message service (SMS) text messaging or social media. To date no large scale randomized effectiveness trials of cessation apps have been published. In contrast to the robust body of literature examining text messaging for smoking cessation [12-30], research on apps has been limited to preliminary pilot studies with small samples or short follow-up periods [11,12,31-33] and protocols of trials in progress [34-37].

Despite the limitations noted above, cessation apps are being developed and sold at a rapid pace [38]. Hundreds of cessation apps are available on the popular iPhone and Android platforms; downloads have exceeded a million per month for some apps [5,8,39]. Consumers are purchasing these apps with the hope of increasing their chances of quitting smoking, but this may not be the case given the apps’ basic designs, lack of theoretical grounding, and failure to include best practice treatment elements. Furthermore, the popularity of these programs clearly speaks to smokers’ interest in these programs, but we know little about what content and design features are most appealing to users [10,22,40]. This is important since appeal may translate to use, and programs must be used to be effective.

Not much is known about treatment provider knowledge, attitudes, and beliefs about smoking cessation apps. For example, do providers think these tools hold promise for treatment? Will they recommend them to clients/patients? What content and features do they believe are most important? The latter is critical because in the absence of empirical data on which content and design features make smoking cessation apps more engaging and effective, it is necessary to rely on alignment of user preferences with the clinical knowledge and practical experience of treatment providers to guide app development. We are unaware of any prior research that has surveyed the relative perspectives of these two key stakeholder groups about these issues. Prior studies have reported on the content of existing cessation apps [5,8,9,41] and the extent to which content is theoretically grounded [39], but none have presented preferred or recommended content from the perspectives of both treatment providers and smokers.

We address this important gap in the literature in this paper. We surveyed clinicians who routinely provide smoking cessation counseling or other nicotine dependence treatment services to assess their knowledge and attitudes about smoking cessation apps as well as their opinions about which key content and design features should be included in the future. We contrast provider perspectives with perspectives of smokers who own and use smartphones and tablets and represent the target audience for smoking cessation apps. These results can more fully inform how best to leverage the technological capacities of mobile devices in future app development to help people stop smoking.

## Methods

### Setting and Review

All research activities were conducted at the Group Health Research Institute and approved by the Group Health Institutional Review Board.

### Participants

Providers were recruited through the professional LISTSERVs of the Society for Research on Nicotine and Tobacco, the Association for the Treatment of Tobacco Use Disorders, and the Society for Behavioral Medicine. Counselors from the largest US tobacco quitline service provider were also invited to participate. Respondents were eligible if they routinely assisted clients/patients to quit smoking and, on average, treated at least 5 smokers every 3 months. Assistance was defined as providing counseling, support and/or pharmacotherapy; persons who simply provided advice to quit and/or treatment referral were not eligible. Participants had the option to receive a $25 Amazon gift card as a thank you or to remain anonymous. Survey completion was limited to one per person and enforced by allowing only one survey submission per IP address. We also monitored names and email addresses provided to ensure there were no duplicates.

Smokers were recruited via online ads (eg, craigslist, SuperSeattleAds, ClassifiedAds), flyers posted around the Seattle area, and invitation letters mailed to members of Group Health Cooperative (a nonprofit health care system in Washington state) who lived in the Seattle area and were likely smokers. Interested smokers were invited to provide feedback on their attitudes and preferences for mHealth smoking cessation tools. Individuals were eligible if they were at least 18 years old, a current smoker interested in quitting smoking or actively trying to quit, could read and write in English, and owned a smartphone or tablet computer which they used to access the Internet at least occasionally. Eligible smokers attended a one-time focus group during which they completed a written survey and were asked to react to a hypothetical smoking cessation app. Data presented in this paper were collected via the written survey; qualitative reactions to the hypothetical app are presented elsewhere and not discussed here. Smokers received $50 for their participation.

### Survey

Both participant groups provided information about their demographics, education, smartphone use, and use of mobile devices and apps. We also surveyed provider attitudes and beliefs about smoking cessation apps and their comfort using smoking cessation apps and electronic communication with clients/patients using a 5-point scale (completely disagree, somewhat disagree, neutral, somewhat agree, and completely agree). Finally, both providers and smokers rated the importance of 21 hypothetical app design features. Features were categorized into the following domains: cost, reputation, privacy and security, content and user experience, and communication with others. Individual features were chosen to (1) reflect technology-based strategies for implementing best practice treatment recommendations (eg, addressing use of pharmacotherapy, providing social support, and offering cognitive behavioral–based content), (2) reflect ways to leverage other smartphone capacities to make these programs more engaging (eg, gaming), (3) assess perceived limitations of mHealth tools (eg, security and privacy), or (4) understand other user preferences which may inform future program development (eg, cost, reputation). Each feature was rated using a 4-point Likert scale (not at all important, somewhat important, very important, and extremely important). Providers were asked to rate how important each feature would be to them as a clinician if they were recommending a cessation app to their clients/patients. Smokers were asked to rate the importance of each feature if they were considering downloading or using a smoking cessation app.

Provider data were collected online with SurveyMonkey online survey software between June and July 2015. Smokers were surveyed in person in June 2015. All participants provided informed consent and could choose to decline to respond to any survey item.

### Analysis

Descriptive statistics were calculated with Excel 2011 (Microsoft). Comparisons between ratings reported by providers and smokers were analyzed in R version 2.15.1 statistical computing software (The R Foundation) with Mann Whitney U tests. We report mean ratings and *P* values for group comparisons. All respondents were included in the sample denominators, but participants who declined to respond to individual items were excluded from the group comparisons as noted in table footnotes.

## Results

### Provider Characteristics

Of the 344 providers responding to the survey, 264 were eligible and were included in the respondent sample. Providers ranged in age from 21 to 85 years (mean 44, SD 13). They were predominantly female (203/264, 76.9%), white (211/264, 79.9%), and not Hispanic or Latino (239/264, 90.5%). Of the providers responding, 30.7% (81/264) had a doctorate degree (MD, PhD, PsyD, PharmD), 37.5% (99/264) had an advanced practice degree (MA, MS, ARNP, PA), 26.9% (71/264) had a college degree, and 3.0% (8/264) had a high school degree. The vast majority (244/264, 92.4%) had received formal training in nicotine dependence treatment; 73.1% (193/264) considered themselves very knowledgeable about best practice smoking cessation treatment, and 25.0% (66/264) considered themselves somewhat knowledgeable. Practice settings included a tobacco quitline service provider (79/264, 29.9%), primary care setting (58/264, 22.0%), specialty medical care setting (34/264, 12.9%), mental health clinic (29/264, 11.0%), pharmacy (1/264, 0.4%), and other settings as specified by respondents (59/264, 22.3%). The latter predominantly included inpatient, outpatient, and work-place practice settings. Most owned smartphones (231/264, 87.5%) and had previous experience downloading apps (230/264, 87.1%).

### Smoker Characteristics

A total of 40 current smokers were surveyed (average cigarettes per day, 12; SD 12). Participants were predominantly white (25/40, 63%) and not Hispanic or Latino (36/40, 90%); half were female. Smokers ranged in age from 20 to 58 years (mean, 38; SD 12). Most did not have a college degree (26/40, 65%), and 70% (28/40) had an annual household income of $50,000 or less. Of the 40 smokers surveyed, 60% (n=24) owned an Android phone, 30% (n=12) an iPhone, and 10% (4/40) another brand of smartphone. The most common tablet computer owned was an iPad (7/40, 18%), but 55% (22/40) did not own a tablet. More than half (24/40, 60%) primarily accessed the Internet via their smartphone. Nearly half (19/40, 48%) had downloaded a health-related app to their phone, and 13% (5/40) had downloaded a smoking cessation app. Among those who had not downloaded a smoking cessation app, 83% (29/35) said they would consider doing so.

### Provider Attitudes and Beliefs About Smoking Cessation Apps

Provider attitudes and beliefs about mHealth cessation apps are summarized in Table 1. Most agreed (somewhat or completely) that mHealth apps hold promise for helping people quit smoking (203/264, 76.9%) and would recommend them to their clients (201/264, 76.1%), especially if the program were empirically validated (236/264, 89.4%). Most respondents also agreed they would use an app that allowed them to track their clients’/patients’ progress (187/264, 70.8%). Relatively few respondents agreed that effective cessation apps currently exist (112/264, 42.4%) or that there is good empirical evidence that apps can help people quit smoking (81/264, 30.7%).

**Table 1:** Provider attitudes and beliefs about smoking cessation apps (N=264).

	Completely disagree^a^ n (%)	Somewhat disagree^a^ n (%)	Neutral^a^ n (%)	Somewhat agree^a^ n (%)	Completely agree^a^ n (%)
Many of my clients or patients use mHealth apps to manage their health.	23 (8.7)	88 (33.3)	65 (24.6)	74 (28.0)	4 (1.5)
mHealth apps hold promise as a tool to help people stop smoking.	2 (0.8)	10 (3.8)	38 (14.4)	126 (47.7)	77 (29.2)
There is good empirical evidence that stop-smoking apps can help people quit.	26 (0.3)	30 (11.4)	134 (50.8)	69 (26.1)	12 (4.5)
As a clinician, I would recommend a stop-smoking app to my patients or clients trying to quit.	2 (0.8)	12 (4.5)	38 (14.4)	137 (51.9)	64 (24.2)
Effective stop-smoking apps are widely available for smokers.	11 (4.2)	46 (17.4)	84 (31.8)	90 (34.1)	22 (8.3)
If there were an app that allowed me to track my client/patients’ progress quitting smoking, I would use it as a clinician.	10 (4.2)	21 (8.0)	35 (13.3)	113 (42.8)	74 (28.0)
If there were an empirically validated stop-smoking app, I would recommend it.	5 (1.9)	2 (0.8)	10 (3.8)	69 (26.1)	167 (63.3)

^a^Nonresponders ranged from n=10 to n=13 across items and are not reflected in table.

Table 2 summarizes provider comfort using electronic communication with clients/patients. Less than half agreed (somewhat or completely) they were comfortable exchanging text messages or emails with their smoking clients/patients (95/264, 36.0%). However, the majority agreed they would be comfortable communicating electronically if it were through a secure system compliant with the Health Insurance Portability and Accountability Act (HIPAA) (221/264, 84%).

**Table 2:** Provider comfort using cessation apps and electronic client/patient communication (N=264).

	Completely disagree^a^ n (%)	Somewhat disagree^a^ n (%)	Neutral^a^ n (%)	Somewhat agree^a^ n (%)	Completely agree^a^ n (%)
I am comfortable exchanging text messages or emails with my patients/clients.	68 (25.8)	46 (17.4)	45 (17.0)	53 (20.1)	42 (15.9)
I would be comfortable communicating electronically with my patients/clients if it were through a secure HIPAA-compliant system.	7 (2.7)	8 (3.0)	18 (6.8)	98 (37.1)	123 (46.6)

^a^Nonresponders (n=10) are not reflected in table.

### Perceived Importance of Select Features for Smoking Cessation Apps

**Figure 1:**
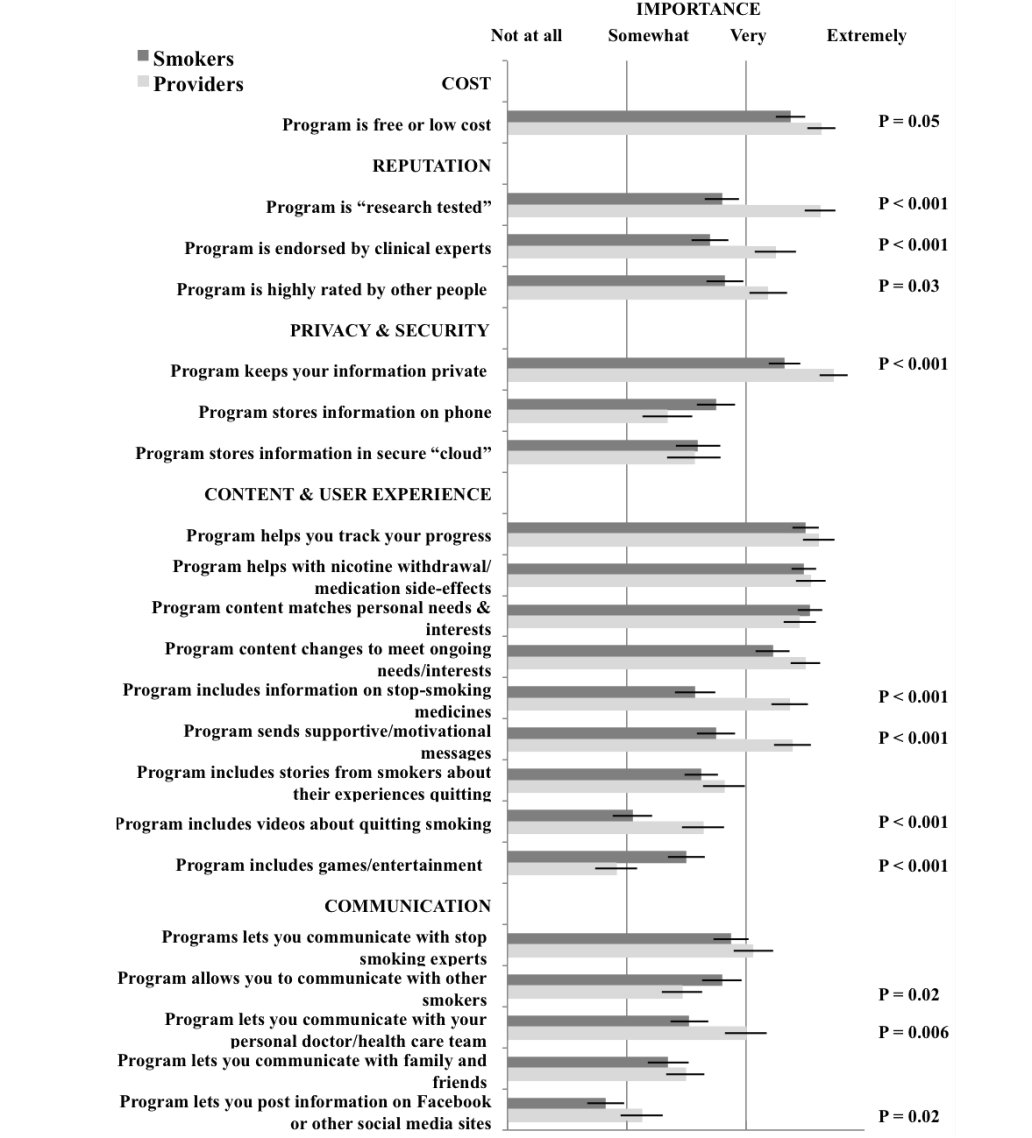
Comparison of providers' and smokers' ratings of important features.

Figure 1 compares providers’ and smokers’ ratings for importance of 21 potential content and design features for smoking cessation apps. All features except gaming and social media were considered at least somewhat important by both groups.

While the mean ratings differed between providers and smokers for many features, on average both groups agreed that the following were very to extremely important as evidenced by a mean rating of 3.0 or greater: the program is free or low cost (providers, 3.6 [SD 0.6] vs smokers, 3.4 [SD 0.8]; *P*=.05), the program keeps your information private (3.7 [SD 0.5] vs 3.3 [SD 0.8]; *P*<.001), the program content matches individual needs and interests (3.5 [SD 0.6] vs 3.5 [SD 0.6]; *P*=.36), the program content adaptively changes as one’s needs and interests change (3.5 [SD 0.6] vs 3.2 [SD 0.9]; *P*=.11), the program helps with managing nicotine withdrawal symptoms and medication side effects (3.5 [SD 0.6] vs 3.5 [SD 0.6]; *P*=.49), and the program allows users to track their progress (3.6 [SD 0.6] vs 3.5 [SD 0.7]; *P*=.32). Although both groups considered programs that are low cost and keep information private important, providers rated their importance significantly higher than smokers.

Both groups considered gaming features or connecting with social media such as Facebook less important than the other features, but the relative importance between groups based on their mean scores differed. Smokers were more likely than providers to rate games or entertainment features at least *somewhat* important (2.5 [SD 1.0] vs 1.9 [SD 0.8]; *P*<.001), but providers were more likely to rate social media connectivity at least *somewhat* important (2.1 [SD 0.8] vs 1.8 [SD 1.0]; *P*=.02) and to believe it is important to include videos about quitting smoking (2.6 [SD 0.8] vs 2.1 [0.9], *P*<.001). Both groups also agreed it was important that apps allow smokers to communicate with experts about their progress (3.1 [SD 0.8] vs 2.9 [SD 0.9]; *P*=.30), allow communication with family and friends about their progress (2.5 [SD 0.8] vs 2.4 [SD 1.1]; *P*=.35), and include stories from other smokers’ experiences quitting (2.8 [SD 0.8] vs 2.6 [SD 0.9]; *P*=.16). The groups also agreed it was at least *somewhat* important that apps store information directly on the smartphone (2.3 [SD 1.0] vs 2.8 [SD 1.0]; *P*=.20) or in a secure cloud (2.6 [SD 1.1] vs 2.6 [SD 1.1]; *P*=.89), but no clear preference was expressed for one over the other in either participant group.

Providers and smokers largely disagreed on the importance of the remaining features. For example, providers thought it was more important than did smokers that apps be highly rated by users (3.2 [SD 0.7] vs 2.8 [SD 0.9]; *P*=.03), endorsed by clinical experts (3.2 [SD 0.8] vs 2.7 [SD 1.0]; *P*<.001) and research tested (3.6 [SD 0.6] vs 2.8 [SD 0.9]; *P*<.001). The providers also believed it was more important that these programs provide supportive motivational messages by text or email (3.4 [SD 0.7] vs 2.8 [SD 1.0]; *P*<.001), include videos about quitting smoking (2.6 [SD 0.8] vs 2.1 [SD 0.9], *P*<.001), include information on stop-smoking medications (3.4 [SD 0.7] vs 2.6 [SD 1.8]; *P*<.001), and allow users to communicate with one’s personal doctor or health care team (3.0 [SD 0.8] vs 2.5 [SD 1.0], *P*=.006). In contrast to providers, smokers said it was more important that they are able to communicate with other smokers about their progress (2.5 [SD 0.8] vs 2.8 [SD 1.0]; *P*=.02) and that programs include games or entertainment (1.9 [SD 0.9] vs 25 [SD 1.0]; *P*<.001); but smokers did not rate either of these features highly.

## Discussion

### Principal Findings

This paper represents the first known survey of smoking cessation treatment providers and smokers to assess and compare their attitudes and opinions about important design features for future smoking cessation apps. While smokers represent the target user group for these programs, clinicians understand the complexities of nicotine dependence and the treatment components this requires. Thus, it is important for mHealth designers and developers to take into consideration the perspectives of both groups when developing smoking cessation apps.

We found that both smokers and clinicians are receptive to using or recommending smoking cessation apps, although clinicians were more open to recommending programs that have been empirically proven to be effective. Smokers rated it as less important that a program be research tested and were also less concerned about the reputation of a program as indicated by user ratings or endorsements by clinical experts. This appears consistent with prior research showing nearly 78% of smoking cessation app users did not check on the credibility of the app publisher before downloading it [42]. The latter may also explain the popularity of existing commercially available cessation apps in the absence of sound empirical support for these programs. Nevertheless, empirical validation is important for smoking cessation apps since these are fundamentally treatment programs.

Features that are low cost, match their personal needs and interests, and help them address nicotine withdrawal and medication side effects and track their progress are rated most highly by smokers. Participants were not asked to delineate what these features mean to them, but matching content to personal needs suggests that adaptively tailored features will help them cope with physiological or environmental cues to smoke. Tracking progress typically takes the form of tracking cigarettes smoked but could also include calculating money saved by not smoking.

Providers rated cost, assistance with withdrawal symptoms and medication side effects, and personalization as important, but they rated privacy as the most important feature for cessation apps. This is not surprising given clinicians’ need to comply with federal privacy laws such as HIPAA. It is notable, however, that 84% said they would be comfortable communicating with clients/patients through a secure, HIPAA-compliant system if it were integrated into the app. Further, both smokers and providers rated the ability to communicate with stop-smoking experts through the program as fairly important (3.1 vs 2.9, *P*=.30). While most mHealth cessation apps do not currently provide this functionality, this evidence supports allowing smokers to have bidirectional communication with stop-smoking experts using systems designed to be HIPAA-compliant. From a technical standpoint, this can be accomplished via a hybrid app which allows users to log into a secure Web portal through the app interface. Information can then be shared, accessed, and stored in the Web portal as opposed to being resident on the mobile device or in an email message. We are currently testing this functionality in our research and expect to see more apps using secure messaging in the future to encourage communication between patients and care providers.

Also of note, smokers seemed to favor communication with tobacco treatment specialists (2.9) over communication with their own health care providers or health care team (2.5), but the ability to communicate with treatment specialists (2.9) was only rated slightly higher than the ability to communicate with other smokers (2.8).

Perhaps as interesting as what providers and smokers rated as most important are the features they viewed as least important: games/entertainment; videos about quitting smoking; communication with friends, family, personal doctor, and health care team; and social media connectivity. Providers tended to give higher ratings, but neither group considered the features critical. These findings may be a cautionary tale for developers. For example, many researchers and developers are working on apps that focus on social media or “gamify” the process of quitting smoking. While these features leverage the technological capabilities of mHealth devices and may appeal to subpopulations of game or social media users and may be novel, they may not ultimately be the most appealing features to smokers on a population level or be viewed favorably by content experts who are in a position to recommend programs to their clients/patients.

Information on stop-smoking medications was not rated highly by smokers but was considered very important by providers. Appropriate pharmacotherapy is a critical component of any best-practice, comprehensive treatment program for nicotine dependence [6,43], and developers are advised to place greater weight on the feedback of providers when considering this feature.

### Strengths and Limitations

This work has several notable strengths. To our knowledge, this is the first research to survey clinical providers’ and smokers’ opinions about smoking cessation apps and one of the first to assess user-centered preferences for smoking cessation apps as opposed to other forms of mHealth intervention (ie, via SMS text messaging or social media platforms). By presenting and contrasting smokers’ and clinicians’ opinions, we provide a richer insight into the relative importance of the reviewed design features than might be gleaned if either group were surveyed in isolation. Another important contribution of this study is that it describes the importance to users of existing design features relative to features that may be considered in future apps. Next, our provider group includes clinicians ranging from physicians, psychologists, nurses, and pharmacists with advanced specialty training to lay counselors working at tobacco quitlines or community-based stop smoking programs. As such, the perspectives may be more representative of the overall provider community than if we had focused on doctorate or masters level professionals only.

This research has several limitations. Our sample reflects the opinions of US treatment providers and may not be generalizable to other populations. Second, it is not clear whether the opinions of the smokers in our sample will generalize to other smokers (US or non-US) because the sample is relatively small (n=40). However, the sample demographics reflect a lower socioeconomic status, racially diverse group of US smokers who were interested in quitting smoking, so the findings are expected to generalize best to this important target group for intervention.

### Conclusion

In summary, smoking cessation apps hold great promise as intervention tools, but current programs are not designed to reflect best practice treatment or take advantage of the full technical capabilities of smartphones and tablet computers. Addressing the latter could make these tools more engaging and more effective. The feedback provided by smokers and providers in this paper offers insight into which content and features researchers and developers should consider in the future. Features rated highly by both groups should receive particular attention, as they are informed by both clinical expertise and user preference. Differences of opinion are notable, as well. In this case, developers should balance user preferences with provider knowledge of best practice treatment. This paper offers insights into this important area of research.

## Abbreviations

HIPAA: Health Insurance Portability and Accountability Act
mHealth: mobile Health
SD: standard deviation
SMS: short message service
